# Salivary Exosome and Cell-Free DNA for Cancer Detection

**DOI:** 10.3390/mi9070340

**Published:** 2018-07-04

**Authors:** Kyung-A Hyun, Hogyeong Gwak, Jaehun Lee, Bongseop Kwak, Hyo-Il Jung

**Affiliations:** 1 School of Mechanical Engineering, Yonsei University, 50 Yonsei-ro, Seodaemun-gu, Seoul 03722, Korea; hyunkkuplus@gmail.com (K.-A.H.); kirapi83@gmail.com (H.G.); 2 Daegu Research Center for Medical Devices and Rehab. Engineering, Korea Institute of Machinery and Materials, 330 Techno Sunhwan-ro, Yuga-myeon, Dalsung-gun, Daegu 42994, Korea; ljh4235@kimm.re.kr

**Keywords:** saliva, circulating biomarker, exosome, cfDNA, pretreatment of saliva, microfluidics

## Abstract

Liquid biopsies are easier to acquire patient derived samples than conventional tissue biopsies, and their use enables real-time monitoring of the disease through continuous sampling after initial diagnosis, resulting in a paradigm shift to customized treatment according to the patient’s prognosis. Among the various liquid biopsy samples, saliva is easily obtained by spitting or swab sucking without needing an expert for sample collection. In addition, it is known that disease related biomarkers that exist in the blood and have undergone extensive research exist in saliva even at a lower concentration than the blood. Thus, interest in the use of saliva as a liquid biopsy has increased. In this review, we focused on the salivary exosome and cell-free DNA (cfDNA) among the various biomarkers in saliva. Since the exosome and cfDNA in saliva are present at lower concentrations than the biomarkers in blood, it is important to separate and concentrate them before conducting down-stream analyses such as exosome cargo analysis, quantitative polymerase chain reaction (qPCR), and sequencing. However, saliva is difficult to apply directly to microfluidics-based systems for separation because of its high viscosity and the presence of various foreign substances. Therefore, we reviewed the microfluidics-based saliva pretreatment method and then compared the commercially available kit and the microfluidic chip for isolation and enrichment of the exosome and cfDNA in saliva.

## 1. Introduction

Liquid biopsy, which allows access to cancer biomarkers in a minimally invasive manner, is an emerging concept for cancer diagnosis and prognosis. Repeated biopsies are essential for cancer patients to monitor cancer progression and establish treatment directions. However, in the case of cancers such as brain, bone, and pancreatic cancer, these repeated tissue biopsies are very painful for the patient and sometimes cause death of the patient. Cancer-derived materials invade adjacent body fluids, including blood, urine, saliva, tears, breast milk, and cerebrospinal fluid (CSF). The materials can also reach body fluids that are distant from the primary tumor by fluid circulation [1]. Although blood is still the major source for the diagnosis and prognosis of cancer, other body fluids are also useful for examining cancer status [2].

Among body fluids, saliva has the advantages of non-invasive acquisition and avoiding personal privacy problems during sampling, unlike urination. Moreover, saliva exhibits various extents of plasma while having unique characteristics like concentration of proteins [3]. Salivary flow is a continuous secretion process from the three bilateral pairs of major salivary glands (parotid, submandibular, and sublingual) and hundreds of minor salivary glands. In general, 1–1.5 L of saliva is produced daily in most adults. Saliva is composed of 99% water and 1% of other substances including electrolytes, proteins (e.g., hormones, enzymes, cytokines, mucins, and immunoglobulins), nucleic acids, exosomes, microorganisms, and cellular debris [4]. The saliva can be harvested in large quantities through stimulation with gum or citric acid, but constituents of stimulated saliva is very complicated depending on the situation. Since the activated salivary glands differ depending on the kind of stimulus, the composition of the saliva, such as the protein concentration and type, is different and it is difficult to specify the standard. Saliva collection methods include spitting and biting a swab that adsorbs the saliva. Spitting is not a suitable method to quantitatively collect saliva and it is difficult to detect analytes by applying the sensor directly to the saliva sample because bubbles take up most of the saliva volume. The swab-based method enables the quantitative collection of saliva even from a person who cannot spit, such as an infant, although loss of the analyte can occur when the saliva is squeezed from the swab.

**Figure 1:**
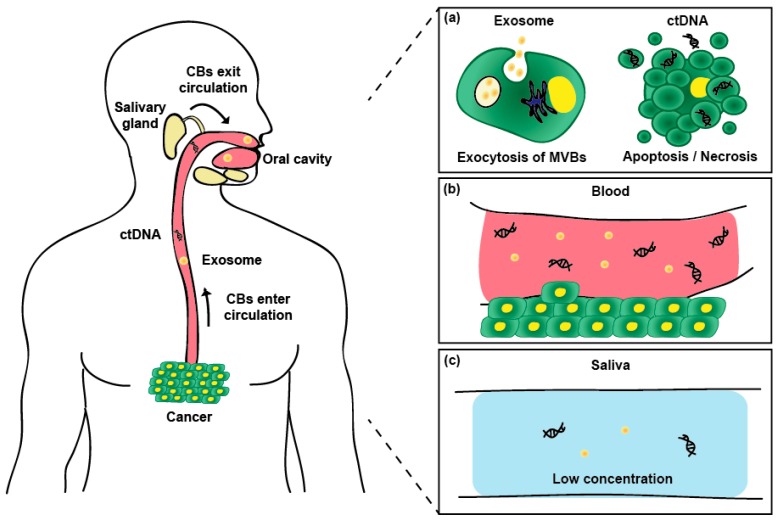
Generation and circulation of salivary circulating biomarkers (CBs). (**a**) Exosome and ctDNA are generated by exocytosis of multi-vesicular bodies (MVBs) and apoptosis and necrosis of cancer cells, respectively; (**b**) The produced biomarkers invade into blood vessels near the cancer tissue; (**c**) By circulating of body fluid, the biomarkers are present in saliva at low concentrations.

Exosomes and cell-free DNA (cfDNA) contain patient-derived information and are discovered in all body fluids such as saliva, blood, urine, tears, breast milk, semen, and spinal cord fluid (Figure 1) [5]. Among these fluids, the salivary exosomes and cfDNA have the attractive advantage of non-invasive acquisition by a simple collecting procedure from the tongue [6]. No expertise is required to collect these samples, and sample collection is easy, even for patients with acquired immune deficiency syndrome (AIDS) or hemophilia. Therefore, the separation and analysis trend of circulating biomarkers is expected to change from blood-based to saliva-based.

In this review, we introduce pretreatment techniques for handling saliva in a microfluidic chip and discuss the importance of exosome and cfDNA analysis using saliva. In addition, we shed light on the research potential of salivary exosome and cfDNA by discussing various approaches to microfluidic based circulating biomarkers isolation in blood, since the microfluidic approach targeting salivary circulating biomarkers has not yet been published as far as we know.

## 2. Techniques for Pretreatment of Saliva

Saliva appears clear and has a simpler composition than blood; therefore, handling of saliva samples for biosensor applications would be expected to be uncomplicated. However, the use of untreated saliva directly on a biosensor can cause fouling of the sensor system and experimental errors, because whole saliva is very viscous and may consist of unpredictable particles, such as food residue.

**Figure 2:**
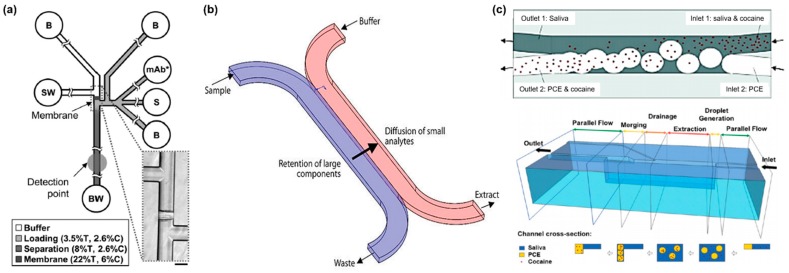
Microfluidic devices for saliva pretreatment. (**a**) The microchip electrophoretic immunoassay (μCEI) device. Fluid wells are labeled according to contents as follows: S, sample; B, buffer; SW, sample waste; BW, buffer waste; mAb*, fluorescently labeled monoclonal antibody to MMP-8. %T and %C refer to the percentage of total acrylamide and bis-acrylamide cross-linker, respectively. The inset shows a 40× bright-field image of the size-exclusion membrane. (Scale bar, 100 μm.) (**b**) Schematic of an H-filter. The H-filter channel is designed to enable large contact between two fluid streams, moving small molecules from the complex sample fluid into the buffer stream. (**c**) (Top) The concept of the droplet-based liquid−liquid extraction method. (Bottom) Three-dimensional schematic of the microfluidic system and below, in parallel, the vertical cross-section views of the channel. Reproduced from ref. [7,9,10] with permission from 2007 National Academy of Sciences, 2006 Nature Publishing Group, and 2013 ACS Publications.

Herr et al. developed a saliva pretreatment method (filtering, enrichment, and mixing) integrated with a microfluidic-based electrophoretic immunoassay (μCEI) to detect oral disease-related biomarkers in saliva (Figure 2a) [7]. This device consists of three major functional regions: (1) a larger pore-size loading gel; (2) a size-exclusion membrane; and (3) a small pore-size separation gel. Saliva sample and detection mixture are electrophoretically introduced into the device through the large pore-size loading gel. Since the polyacrylamide gel is filled in all the channels, electrophoretic transport is dominant in the mobilization of an analyte. The size-exclusion membrane eliminates a wide range of proteins that exceed 10 kDa in size. Passing through these channels, the saliva and detection mixture are mixed and the target analytes combined with the detection molecule are enriched. The gel composition of the separation channel provides enhanced size-based sieving of analytes that are very similar in their charge-to-mass characteristics, as is the case with the target analyte and detection molecule complex. Using the μCEI chip, the authors successfully measured the concentration of endogenous matrix metalloproteinase-8 in saliva, which is a major tissue destructive enzyme in periodontal disease.

Pretreatment methods based on filtration are widely used for the removal of various non-target proteins and particles but still require degradation of mucins to reduce the viscosity of saliva, usually using an enzyme. The viscoelastic behavior of saliva derives from the mucins (glycoproteins), which have high molecular weights and can self-aggregate to form very large structures. Yager and coworkers reported a microfluidic device (H-filter) to reduce saliva viscosity and propensity to foul the biosensor (based on surface binding), while maintaining the concentration of target molecules [8]. The authors devised a two-step sample preconditioning method. The first step was membrane filtration to remove cells, large debris, and the majority of the mucin glycoproteins. The second step prevented biosensor fouling due to unwanted proteins by using the H-filter to extract target molecules from filtered saliva based on diffusion in laminar flow (Figure 2b) [9]. This two-step conditioning protocol removed 97% of glycoproteins and 92% of unwanted proteins, while retaining 23% of target molecules in the patient’s saliva sample. Moreover, the surface fouling decreased 3.6-times less than the unprocessed saliva. The H-filter is the standard method for microfluidic-based extraction. Since the flow in the H-filter is laminar, the liquid can mix only by diffusion through the parallel flow lines in the channel. However, the particles are able to move from one parallel liquid stream to the other. Applying a droplet-based approach to the H-filter could increase the diffusion interface because the droplet acts the particle. Using this approach, de Rooij and coworkers devised a microfluidic droplet-based liquid–liquid extraction chip to detect cocaine in saliva (Figure 2c) [10]. At first, in the microfluidic channel, droplets of the acceptor phase organic solvent tetrachloroethylene, also termed perchloroethylene, encapsulate the cocaine in the donor phase saliva. The droplets containing cocaine merged again to a continuous phase for the infrared-light-absorbing based detection. Compared with microfluidic-based H-filters, de Rooij and coworkers reported the extraction of target molecules with at least two-times higher yield.

Most studies of saliva pretreatment have focused on optimization of saliva conditioning for detection of proteins. Commercially available kits operate in this manner. This approach is difficult to apply to sample pretreatment for detection of exosome and cfDNA in saliva. For example, filtration methods that reduce the viscosity of saliva by removing glycoproteins can also result in the loss of exosomes because exosomes are larger than general proteins. Therefore, it is necessary to study saliva pretreatment methods that are optimized to detect and analyze exosomes and cfDNA in saliva.

## 3. Salivary Exosome and Cell-Free DNA

The exosome is an extracellular vehicle 30–150 nm in size produced during cell metabolism. Exosomes are biologically stable and contain a great deal of bio-information for cancer diagnosis, including DNA, RNA, protein, and various biomarkers [11,12]. The surface of an exosome contains specific biomarkers, such as cluster of differentiation CD9, CD63, and CD81, and tumor susceptibility gene (TSG)101, which can be used for cancer diagnosis [13]. Moreover, the exosome contains cancer patient-specific genes that allow personalized chemotherapy. Karolina et al. reported the presence of the oncogenic mutation biomarkers phosphatidylinositol-4,5-bisphosphate 3-kinase catalytic subunit alpha (PIK3CA), Kirsten rat sarcoma viral oncogene homologue (KRAS), v-Raf murine sarcoma viral oncogene homolog B (BRAF), and tumor suppressor p53 (TP53) in salivary exosomes of cancer patients [14]. Various genotyping methods are widely used for the analysis of salivary exosomes, such as polymerase chain reaction (PCR) and flow cytometry combined beads, emulsion, amplification, magnetics (BEAMing) [15], real-time quantitative reverse transcription PCR [16], droplet-based digital PCR [17], and next-generation gene sequencing (NGS) technologies [18]. Yoshioka et al. [19] reported that the CD147/CD9 expression in exosomes differs significantly between healthy individuals and colon cancer patients using a novel system (ExoScreen). ExoScreen can detect specific proteins on exosomes based on the fluorescence intensity changes caused by the interaction of these proteins in donor and acceptor beads. The authors described that the CD147/CD9 levels in healthy donors were lower than those in colorectal cancer patients, whereas CD147/CD9 levels gradually increased in patients with cancer stage progression from 1 to 4.

The cfDNA, 150–200 base pair (bp) in size, is an important biomarker [20]. Cancer cells have a higher metabolic rate than normal cells, resulting in increased apoptosis and necrosis. Therefore, cfDNA levels in cancer patients are higher than those in healthy people in general [21]. In this process, a large quantity of cfDNA is produced and enters body fluids. Since the cfDNA is biodegraded by DNase faster than exosomes or protein-based biomarkers [22], it reflects the most recent patient status. Among the various cfDNAs, the cancer-specific gene called circulating tumor DNA (ctDNA) is an important biomarker to analyze the status of cancer. ctDNA accounts for a small percentage of the total cfDNA (0.01–1%), but provides information of genetic mutations in cancer patients. The 5-year survival rates of ctDNA-detected and non-ctDNA cancer patients were reported as 33% and 65%, respectively [23], indicating that the level of ctDNA as well as the genetic information it harbors can be used to predict the survival rate of cancer patients. The ctDNA concentration and DNA sequencing analysis are essential for patient treatment due to the individual cancer patients’ specificity of ctDNA. Techniques for detecting ctDNA include BEAMing [24,25], TAm-seq (Tagged-amplicon deep sequencing), digital PCR [26,27], and whole genome sequencing (WGS) [28,29]. Thierry et al. studied KRAS and BRAF mutations in a cfDNA analysis of 106 colorectal cancer patients. The BRAFV600E mutation showed 100% specificity and sensitivity, and the KRAS point mutation showed 98% specificity and 92% sensitivity [30].

## 4. Applicable Technologies to Enrich and Isolate Exosome and Cell-Free DNA

Since the salivary exosome and cfDNA are mixed with various biomarkers and are present at a concentration approximately 1000-fold lower than that of biomarkers in blood, their isolation and enrichment is crucial for successful down-stream analysis [31]. The many commercially available exosome isolation kits are usually based on immune-affinity capture, size exclusion chromatography (SEC), and precipitation. We have tabulated these kits considering features that focus on their use with saliva (Table 1). Despite the growing interest in cfDNA, most commercially available cfDNA isolation kits are based on non-cfDNA specific isolation methods, such as separation by silica adsorption. Since cfDNA is a much smaller fraction than the genomic DNA from blood cells, contamination of genomic DNA causes analytical errors like false positive results. Several recent kits specifically capture the cfDNA. The polymer mediated enrichment (PME) technology (Analytic Jena) captures cfDNA by encapsulating it with the PME polymer. The collisions are collected by centrifugation and the captured cfDNAs are dissolved in a specific buffer. The cfPure™ cell-free DNA purification kit (Amsbio) uses silica-coated paramagnetic particles with a specific buffer optimized for efficient recovery of 100–500 bp DNA fragments. However, most cfDNA specific purification kits, including the aforementioned, do not adequately describe how to capture the cfDNA specifically due to patent-related proprietary concerns. Thankfully, several papers have compared the performance of cfDNA purification kits using blood samples [32]. However, no comparative analysis focused on the use of saliva samples has been performed. The commercial kits have short processing times and have made no use large equipment like ultracentrifuges. However, the kits are still prone to loss of the target during the multiple steps. The procedures are also very labor-intensive due to their complexity.

**Figure 3:**
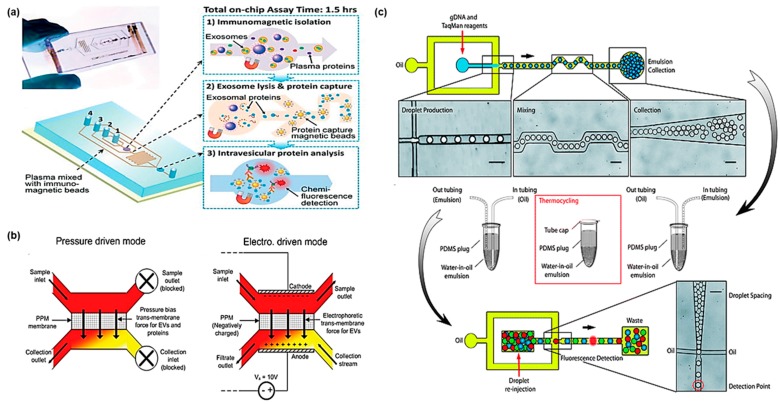
Microfluidic devices for exosome and cell-free DNA research. (**a**) Multistage exosome microfluidic for immunomagnetic isolation of exosome, exosome lysis, protein capture, and intravesicular protein analysis; (**b**) PPM membrane based microfluidic system for exosome filtration; (**c**) Droplet-based microfluidic device for detecting mutated DNA in a quantitative manner. Reproduced from ref. [33,37,39] with permission from 2014 Royal Society of Chemistry, 2012 Royal Society of Chemistry, and 2011 Royal Society of Chemistry.

Microfluidic and lab-on-a-chip technologies can provide an alternative that can automate the process as well as reduce the time and labor cost. Microfluidic platforms have several advantages compared to the commercialized isolation kits, such as high throughput, sensitivity, purity, and low cost. By analyzing exosomes and cfDNA through microfluidics technologies, clinical cancer research may progress more rapidly and efficiently. So far, microfluidic based isolation of saliva exosome and cfDNA have not been published yet. This can be explained by the fact that the researchers are more familiar with the isolation of exosomes and cfDNA from the blood. However, the saliva component is simpler than the blood, so that the microfluidic technique targeting the circulating biomarkers in the blood as described below can be applied to the isolation of exosome and cfDNA in saliva through integration with saliva pretreatment methods mentioned Section 2. Methods for the isolation of exosomes in the blood employed in microfluidics include immuno-affinity [33,34,35], ciliated micropillars [36], porous polymer monoliths (PPM)-based membrane filters [37], functionalized surfaces [38], and droplet-based systems [39,40]. He et al. developed a microfluidic chip that is simultaneously capable of immunomagnetic isolation of exosomes from plasma, exosome lysis, protein capture, and intravesicular protein analysis (Figure 3a) [33]. The exosome can be isolated and enriched through CD9, CD63, CD81, epithelial cell adhesion molecule (EpCAM), alpha-insulin-like growth factor-1 (α-IGF-1R), and cancer antigen 125 (CA125)-coated magnetic beads. The developed chip can handle plasma samples with a throughput of 20 μL/h, where the required sample amount is approximately 100 times less than conventional methods. The developed system has been used to analyze the surface of exosomes in healthy donors and non-small cell lung cancer (NSCLC) cancer patients, with 3.9-fold higher exosomal protein expression in NSCLC patients than healthy donors [33]. The results clearly indicated the analytical capability of the technique for exosome phenotyping and quantitative protein determination. Wang et al. developed a ciliated micropillars microfluidic system that can isolate nanoscale exosomes [36]. Porous silicon nanowires on the surface of the micropillar array can isolate the exosomes with 30–200 nm inter-spacing forest. This system reportedly isolated samples over a short time (approximately 10 min) with 60% purity. However, further optimization is required to improve the purity of recovery. Ryan et al. introduced a microfluidic system that can separate the exosome through a PPM membrane (Figure 3b) [37]. PPM is widely used as a filter membrane for separating small molecules in microdevices, and decreasing porogen content leads to a structurally compact membrane. Two different working modules have been applied in the system: pressure-driven and electrophoresis-driven modules. In the pressure driven mode, PPM filter pore size is the most important factor in separating exosomes. When hydraulic pressure is applied to the PPM membrane, smaller molecules pass through the membrane depending on the pore size. When the porogen concentration of the PPM filter was 58%, 60%, and 62%, the pore size of the filter was 100 nm, 500 nm, and 1000 nm, respectively. The microfluidic chip fabricated with 60% porogen is the most efficient concentration capable of separating exosomes. The pressure-driven mode can obtain exosomes in a short time (approximately 40 min) with a small amount (40 μL) of sample. The electrophoresis driven mode is a method of separating exosomes based on their electrophoretic mobility using a negatively-charged phospholipid membrane. Negatively-charged exosomes are isolated through the PPM membrane by electric force in a device with an electric field of 6.7 V/cm. The electrophoresis mode is capable of selectively removing proteins and extracting higher purity exosomes than the pressure driven mode. The developed device will be able to selectively isolate exosomes of various sizes depending on the pore size and remove non-specific proteins. Thus, it should contribute to studies of specific DNA inside the exosome.

Kanwar et al. developed a microfluidic system called ‘Exochip’ that enables the isolation, on-chip quantification, and molecular characterization of exosomes [38]. The Exochip isolates exosomes through an internal channel coated with CD63 antibody and quantifies the fluorescence density of exosomes by fluorescent carbocyanine dye (DiO) staining. Use of the Exochip detected a difference between CD63 and Rab5 protein levels in healthy and pancreatic cancer patients of 3.17-fold and 1.75-fold, respectively. This result indicated that cell apoptosis and necrosis would more likely occur, and exosome levels would be higher, in cancer patients than in healthy individuals. In addition, exosome separation using the Exochip was reported to be 16.2-fold more rapid compared to Exo-spin (10 min versus 162 min). Exochip-mediated isolation and analysis of exosomes was performed using only CD63 antibody. However, various exosome protein biomarkers have been reported, which include CD9 and CD81. Therefore, applying various biomarkers to Exochip will lead to diverse exosome-based cancer research efforts.

Similar to exosomes, cfDNA is also important in the liquid biopsy diagnosis of cancer. Isolating and analyzing cfDNA in the biofluid is an important step for the treatment and prevention of cancer. So far, to our knowledge, there is no microfluidic-based research to specifically isolate and analyze cfDNA even in the blood. However, many studies have explored microfluidic-based genomic DNA isolation and detection. Reedy et al. developed a polymethyl methacrylate microfluidic device capable of purifying DNA samples [41]. The system employed an array of micro-post structures that provide a large surface area. The functionalized micro-post chitosan surface can enable binding of DNA by a charge-charge interaction at pH 5 and release of the DNA at pH 9, resulting in a recovery rate of 47.8 ± 9.3%. Although this platform provides a useful way to purify cfDNAs, the low recovery rate needs to be improved. Digital PCR enables massive numbers of DNA amplifications simultaneously. This approach can examine the ratio of mutated and wild-type sequences in a single target DNA [45]. Pekin et al. reported the quantitative and selective detection of rare mutations of DNA using digital PCR (Figure 3c) [39]. This system can generate 10 pL droplets at 30 kHz (1.8 × 10^6^ droplets/min). After thermocycling amplification, the wild-type and mutated DNA are selectively distinguished by fluorescence analysis. SW48 is a colorectal cancer cell line with wild-type KRAS alleles. Fluorescence analysis by digital PCR showed no KRAS mutation. On the other hand, the SW620 colorectal cancer cell line with the G12V KRAS mutation displayed the KRAS mutation without the wild-type alleles. In addition, the developed platform can perform multiple mutational screenings for G12A, G12C, G13D, and G12S, including the KRAS G12V mutation, clearly demonstrating the DNA analysis capability of microfluidics for rare mutant detections.

There are many microfluidic approaches to isolate exosomes and cfDNA from cancer patients through serum or blood. However, the microfluidic isolation system using salivary exosomes and cfDNA is still in its infancy. The separation of exosome and cfDNA using microfluidics has distinctive advantages. By integrating and optimizing these advantages using microfluidics, researchers will be able to obtain salivary exosomes and cfDNA with higher efficiency than the current technology.

## 5. Conclusions

Presently, cancer patients must undergo serial blood sampling to monitor the progression of the disease and evaluate treatment effectiveness. Although liquid biopsy using blood samples is less painful than tissue biopsy, serial blood sampling is a burden on cancer patients and creates the risk of needle-related infection. Use of saliva eliminates these blood biopsy-related issues. Salivary biomarkers reflect the cancer status of patients. Among the various biomarkers in saliva, exosomes and cfDNA have the potential for real-time monitoring of cancer patients, cancer diagnosis, and prognosis. Analysis of exosomes and cfDNA in saliva using microfluidic chips is still in its infancy. A standard operating procedure of saliva collection, which includes information on the swab material, position of the saliva collector in the oral cavity, and collection time, should be established for the efficient acquisition of exosomes and cfDNA in saliva. Since the concentration of these biomarkers in saliva is about 1000-times lower than that in blood, it is also important to implement a sensitive sensing technology in microfluidic chips. The various advantages of microfluidics include the easy introduction of different technologies into one chip. Thus, a continuous flow from saliva pretreatment to isolation and detection of biomarkers will reduce the loss of exosomes and cfDNA.

## Figures and Tables

**Table 1:** Applicable technologies for isolation of circulating biomarkers in saliva.

Circulating Biomarkers	Commercial Kits		Microfluidic Devices	
	Product	Feature	Techniques	Feature
Exosome	ExoFACS™ (BioVision, Inc., San Francisco, CA, USA)	Immune-based separation (4 μm antibody-coated beads) Suggested volume: 250–500 μL 117 min Using diluted saliva	Immuno-affinity [33]	Immuno-affinity based separation Using CD9, CD63, CD81, EpCAM, α-IGF-1R, CA125 antibody-coated magnetic bead approximately 100 min Using minimal sample volume of 30 μL
	Minute™ (Invent Biotechnologies, Inc., Plymouth, MA, USA)	SDS-PAGE Suggested volume: 100–600 μL 90 min to overnight	Ciliated micropillars [36]	400 nm long porous silicon nanowire-coated micropillars Using minimal sample volume: 10–100 μL Size based separation Retention rate: 60%
	Exo-spin^TM^ (Cell Guidance Systems, Ltd., Cambridge, UK)	Size Exclusion Chromatography (SEC) Suggested volume: 1–50 mL 162 min	PMMA-based membrane filters [37]	Porous polymer monoliths(PPM) filtration Throughput: 2 modes Pressure-driven filtration: 1 μL /min Electrophoresis-driven filtration: 2 μL /min Pressure mode: 40 min Electrophoresis mode: 2 h
	Saliva Exosome Purification Kit (Norgen Biotek, Corp., Thorold, ON, Canada)	Resin based separation Suggested volume: 0.5–2 mL Using diluted saliva 45 min	Functionalized surfaces (Exochip) [38]	CD63 exosome-specific antibody coated Throughput: 50 μL /min Suggested volume: 400 μL 10 min
cfDNA (DNA)	GeneFix^TM^ Saliva DNA isolation kit (Cell projects, Ltd., Harrietsham, UK)	Spin column chromatography Saliva input: 0.5–1 mL Yields in excess of 100 μg from 2 mL of saliva Average purity (OD260/280): >1.8	Chitosan coated PMMA high surface area [41]	Functionalized surface(chitosan) Throughput: 1.6 μL/min Suggested volume: 25 μL Recovery rate: 47.8 ± 9.3%
	Oragene^®^-DNA (DNA Genotek, Inc., Ottawa, ON, Canada)	Ethanol precipitation Saliva input: 0.5 mL Median yield: 1.9–35.1 μg (2 mL of saliva) Average purity (OD260/280): 1.6–1.9	Digital PCR using droplet based microfluidics [39]	Using droplet based microfluidics Droplet volume: 10 pL Generation speed: 30 kHz Multiple mutation can be analyzed 0.01–1.7% of mutation can be detected
	Saliva DNA Isolation Kit (Norgen Biotek Corp., Thorold, ON, Canada)	Spin column chromatography Saliva input: 0.25 mL of fresh saliva, 0.5 mL of preserved saliva Average yield: 3–7 μg (0.25 mL of saliva) Average purity (OD260/280): 1.7–2.1	Tagmentation chemistry and solid phase reversible immobilization (SPRI) based integrated microfluidics [42]	Tagmentation chemistry: Extraction DNA SPRI: Purification DNA Fully automated 96 cases of DNA extraction and purification simultaneously react. Low sample volume required: 36 nL DNA purity: >80%
	Saliva DNA Isolation Kit (BioChain Institute, Inc., Newark, NJ, USA)	Spin column chromatography Saliva input: 50–200 μL Average yield: 0.25–4 μg (200 μL of saliva) Average purity (OD 260/280): >1.8 Total isolation time: <25 min DNA size: 99% of DNA is >20 kb	DNA purification and PCR amplification based integrated microfluidics [43]	Micro-sample processing device (μSPD) DNA purification process: 3 steps (load, wash, elute) DNA amplified on chip (low to high concentration) Low sample required: 12 μL Sample reduction for DNA purification: >25-fold
	Mini·SAL™ Saliva DNA Isolation Kit (Oasis Diagnostics® Corporation, Vancouver, USA)	Spin column chromatography Saliva input: 0.1–1 mL Average yield: 2.21 μg (200 μL of saliva) Average purity (OD260/280): 1.85 Total isolation time: >34 min Average DNA size: approximately 30 kb	DNA extraction, amplification, detection based integrated microfluidics [44]	Monolithic aluminum oxide membrane for DNA extraction: seven parallel reaction wells Possible to real-time PCR on chip using extracted DNA Multiple target DNA is possible to identify Low sample required: 13.5 μL Total analysis time: <2.5 h
